# Cholécystectomie laparoscopique ambulatoire: première expérience en Tunisie

**DOI:** 10.11604/pamj.2017.28.78.9564

**Published:** 2017-09-27

**Authors:** Haithem Zaafouri, Skander Mrad, Nizar Khedhiri, Dhafer Haddad, Ahmed Bouhafa, Anis Ben Maamer

**Affiliations:** 1 Service de Chirurgie Générale, Hôpital Habib Thameur, Tunis, Tunisie

**Keywords:** Cholécystectomie, laparoscopie, ambulatoire, Cholecystectomy, laparoscopy, outpatient clinic

## Abstract

**Introduction:**

La cholécystectomie laparoscopique est le gold standard de la prise en charge des calculs vésiculaires symptomatiques. Il existe une importante controverse quant au fait de savoir si elle devrait être pratiquée en chirurgie ambulatoire ou dans le cadre d'une chirurgie avec hospitalisation d'une nuit pour ce qui concerne la sécurité des patients. Le but du travail est d’évaluer l'impact de la cholécystectomie laparoscopique en chirurgie ambulatoire versus en chirurgie avec hospitalisation d'une nuit sur les critères de jugement axés sur le patient, tels que la mortalité, les graves événements indésirables et la qualité de vie.

**Méthodes:**

Il s’agit d’une étude transversale descriptive réalisée au sein du service de chirurgie générale de l’hôpital Habib Thameur, sur la période allant de Mai 2009 à Février 2010. Cette étude porte sur 67 malades porteurs d’une lithiase vésiculaire symptomatique ayant eu une cholécystectomie laparoscopique en ambulatoire (CLA). Étaient exclus de l’étude: les malades ASA III et IV, les diabétiques sous sulfamides ou sous insuline, les grands obèses, les malades de plus de 65 ans et moins de 18 ans, ceux avec un antécédent de chirurgie abdominale majeure, les malades suspects d’une lithiase de la voie biliaire principale, d’une cholécystite aiguë ou d’une pancréatite. Pour être traité par CLA, le malade devait résider à moins de 50 km de l’hôpital, et avoir la possibilité d’une présence adulte à ses côtés.

**Résultats:**

Dix-sept patients étaient inclus puis exclus de notre étude devant la découverte per opératoire de signes de cholécystite aigue ou devant des difficultés de dissection amenant le chirurgien à mettre un drain de Redon en sous hépatique en fin d’intervention. Finalement, 50 patients ont été retenus: 7 hommes et 43 femmes d’âge moyen de 48 ans. L’intervention se déroulait selon les modalités habituelles. A la sortie de la salle de réveil, le patient était dirigé en secteur ambulatoire où une alimentation liquide était autorisée. Le malade était revu avant 19 h et la sortie décidée si une analgésie orale était possible, si une alimentation liquide était tolérée, s’il n’existait aucun trouble de la diurèse, et si le patient acceptait un retour à domicile avec un traitement antalgique et anti-inflammatoire à la demande. Trente neuf patients (78%) ont quitté l’hôpital et 11 ont été gardés. L’âge > à 45 ans, la durée de l’anesthésie > à 70 minutes et la fatigue post opératoire ont été identifié comme facteur de risque de sorties ratées. Aucune réadmission n’a été observée. Les patients qui ont pu être mis sortants ont été satisfaits du protocole de prise en charge avec des réponses majoritairement de type excellent et bon (94%).

**Conclusion:**

La chirurgie ambulatoire semble tout aussi sûre que la chirurgie avec hospitalisation d'une nuit dans la cholécystectomie laparoscopique avec un faible taux de complication et de réadmission chez des malades sélectionnés, et avec une réduction du coût de l’intervention.

## Abstract (en)

**Introduction:**

Laparoscopic cholecystectomy is the gold standard treatment for symptomatic gallstones. There is some debate as to whether it should be performed in outpatient surgery or in one-day surgery to improve patient safety. This study aimed to evaluate the impact of laparoscopic cholecystectomy performed in outpatient surgery versus one-day surgery on standards such as mortality, serious adverse events and quality of life.

**Methods:**

We conducted a cross-sectional descriptive study in the Department of General Surgery at the Habib Thameur Hospital over the period May 2009-February 2010. We here report 67 cases of symptomatic vesical lithiasis treated with outpatient laparoscopic cholecystectomy (OLC). ASA III and IV patients, diabetic patients treated with sulfonamides or insulin, severely obese patients, patients over 65 years of age and under 18 years of age, patients with a history of major abdominal surgery, patients with suspected lithiasis of the common bile duct, acute cholecystitis or pancreatitis were excluded from the study. Patients had to reside within 50 km of the hospital and be accompanied by an adult to undergo OLC.

**Results:**

Seventeen patients were included and then excluded from our study because of the perioperative detection of signs of acute cholecystitis or difficulties in dissection leading to subhepatic drainage using Redon catheter at the end of the intervention. Finally, our study included 50 patients, 7 men and 43 women; the average age was 48 years. Surgery was based on the most common procedures. After leaving the recovery room, patients were conducted in the outpatient sector where they received a liquid diet. The patients were examined before 7 o’clock in the evening and discharge was established on the basis of the possibility of establishing an oral analgesic treatment, patients tolerance to liquid diet, the lack of urinary disorder, patients acceptance for discharge and analgesic and anti-inflammatory treatment if needed. Thirty-nine patients (78%) were discharged from hospital and 11 were kept in hospital. Patients > 45 years of age, anesthesia duration > 70 minutes and post operative fatigue were identified as risk factors for unsuccessful discharge. No readmission was observed. Discharged patients were satisfied with the therapeutic protocol, resulting in excellent and good outcome in the majority of cases (94%).

**Conclusion:**

Outpatient laparoscopic cholecystectomy seems to be as safe as day surgery laparoscopic cholecystectomy having low rate of complications and of hospital readmissions in some selected patients and lower surgery costs.

## Introduction

La chirurgie ambulatoire a montré ses avantages en termes de bénéfices économiques et de diminution des infections nosocomiales. Elle tend à devenir la norme avec déjà plus de 70% des patients opérés d’une hernie inguinale dans les pays scandinaves et jusqu’à 87% aux Etats-Unis selon cette approche [1, 2]. Une unité de chirurgie ambulatoire (UCA) a été crée au centre hospitalo-universitaire Habib Thameur de Tunis en 2008. Cette UCA était ouverte au début pour la chirurgie pariétale et proctologique, puis pour la cholécystectomie laparoscopique ambulatoire (CLA). L’objectif principal de ce travail était d’étudier à l’issue d’une première année d’exercice en ambulatoire, d’une part la faisabilité et la sureté de la CLA et d’autre part les facteurs d’échec de réalisation de cette chirurgie et les facteurs de réadmission après sortie le jour même.

## Méthodes

Il s’agit d’une étude transversale descriptive réalisée au service de chirurgie générale de l’hôpital Habib Thameur de Tunis, durant la période s’étendant de Mai 2009 à Février 2010.

### Matériels

***Critères d’inclusion:*** Les critères d’inclusion ont été: l’âge supérieur à 18 ans, la classe ASA 1 ou 2, l'accès facile à l’hôpital (grand Tunis et moyens de transports disponibles), un accompagnant adulte disponible pendant les 48 premières heures et enfin, le consentement éclairé du patient.

***Critères de non inclusions:*** N’ont pas été inclus les patients: âgés de moins de 18 ans ou sous tutelle, âgés de plus que 65 ans, de classe ASA3 ou supérieure, diabétiques sous sulfamides hypoglycémiants ou sous insuline, aux antécédents de chirurgie abdominale majeure, présentant une suspicion d’une lithiase de la voie biliaire principale, présentant une suspicion de cholécystite aigue, mis sous traitement anticoagulant au long cours et ayant une notion d’allergie aux drogues anesthésiques utilisées.

***Critères d’exclusion:*** Les patients ont été exclus chaque fois où il y avait, une décision chirurgicale de conversion en laparotomie, ou une constatation per opératoire d’une cholécystite aigue, d’un saignement important ou d’une fuite biliaire ou une nécessité de mettre en place un drain de Redon.

## Méthodes

### Organisation de l’unité ambulatoire

Pour cette étude nous avons utilisé les structures hospitalières dites conventionnelles. En effet tous les patients inclus dans notre étude ont été hospitalisés vers 7 heures, le matin de l’intervention, selon la même procédure administrative que les hospitalisations classiques. Après leurs préparations, ils ont été acheminés vers le bloc opératoire du service, puis une fois l’intervention finie ils ont été surveillés dans la salle de surveillance post interventionnelle (SSPI). Une fois les critères de sortie de la SSPI remplis, les patients ont été transférés au service d’hospitalisation. La procédure de sortie de l’hôpital a été la même que pour les autres patients non ambulatoires mais elle a été faite le jour même, avant 19h.

### Critères de sortie de l’hôpital

Les critères de sortie de l’hôpital ont été: 1) Une stabilité des paramètres vitaux; 2) une analgésie efficace par voie orale exclusive (pas de recours à la voie iv); 3) prise de boissons per os bien tolérée, sans nausées ni vomissements; 4) une déambulation possible (lave-mains, toilette) sans vertiges; 5) une bonne miction spontanée; 6) le patient se sent prêt à rentrer chez lui; 7) l’accord du chirurgien pour la sortie du patient; 8) la sortie est effectuée avant 19h.

### Paramètres analysés

L’analyse a porté sur la réussite ou l’échec de la sortie des patients inclus et non exclus de l’étude, le jour même de l’intervention, définissant ainsi les deux groupes : le groupe « sortants» (les patients qui ont été mis sortants le jour même de l’intervention avant 19h) et le groupe « sorties ratées » (les patients qui ont été hospitalisés jusqu’au lendemain). Nous avons comparé les deux groupes concernant les caractéristiques démographiques, la durée de l’anesthésie, celle de la laparoscopie, la consommation des drogues anesthésiques et d’éphédrine, la douleur post opératoire (DPO), la survenue de nausées et vomissements post opératoires (NVPO), la durée du séjour en SSPI et les plaintes des patients au service. L’analyse statistique a été réalisée à l’aide du logiciel SPSS 17.0 (Statistical Package for the Social Sciences software for Windows). Les variables quantitatives ont été exprimées en moyenne ± écart type alors que les variables qualitatives ont été exprimées en pourcentage (%). Le test t de Student a été utilisé pour comparer les variables quantitatives tandis que le test X2, et si approprié le test de Fisher, ont été utilisés pour la comparaison des variables qualitatives. Le seuil de signification a été fixé à 0,05. L’évaluation après sortie de l’hôpital a été faite le 1^er^ et le 3^ème^ jour postopératoire par un entretien téléphonique, et au 7^ème^ jour postopératoire lors de la 1^ère^ consultation postopératoire. Nous avons évalué la survenue des NVPO, des DPO, de la rétention urinaire et la reprise du transit. Enfin, nous avons enregistré la survenue de morbidités nécessitant la consultation avant la date du rendez-vous postopératoire (à J7 post opératoire) ou la réadmission à l’hôpital.

## Résultats

### Données générales

**Figure 1:**
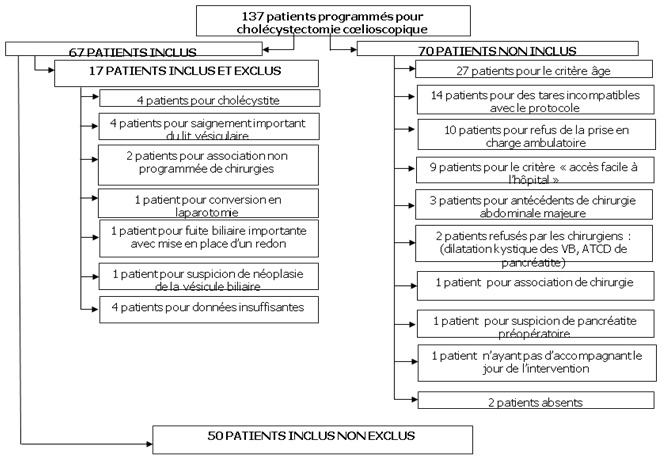
Patients retenus

Cent trente sept patients (30 hommes et 107 femmes avec un sexe ratio à 0,28), devant subir une cholécystectomie laparoscopique (CL) pour lithiase vésiculaire simple symptomatique ont été programmés et vus à la consultation pré-anesthésique et au staff anesthésie-chirurgie. L’âge moyen des patients était de 52 ans± 15. Soixante-dix patients soit 51% n’ont pas été inclus et soixante sept patients soit 49% ont été inclus sur la période d’étude sur un total de 137 patients. Dix sept patients ont été inclus puis exclus. Finalement nous avons retenu comme le montre la Figure 1, 50 patients. Dans le groupe des patients retenus, sur lequel nous avons effectué notre étude statistique, 39 patients (soit 78%) ont pu être mis sortants le jour même de l’intervention « groupe sortants », alors que 11 patients (soit 22%) n’ont pu l’être que le lendemain « groupe sorties ratées ».

### Analyse descriptive

L’âge moyen des patients retenus était de 47 ans (extrêmes allant de 32 ans à 61 ans). L’âge moyen était 43 ± 11 ans dans le groupe « sortants » et 52 ± 9 ans dans le groupe « sorties ratées ». Neuf patients sur 11 du groupe « sorties ratées » avaient un âge > à 45 ans, alors que dans le groupe « sortants » ils étaient 6/39. L’âge > à 45 ans a été identifié comme facteur de risque de sorties ratées avec une valeur de p à 0,000 qui est statistiquement significative. Sept hommes et 43 femmes, ont été retenus : deux hommes dont le groupe « sorties ratées» et un homme dans le groupe « sortants ». Le sexe masculin n’a pas été retenu comme facteur de risque de sorties ratées (p= 0.1). Les patients inclus avaient des BMI moyen de 29 ± 4 avec un minimum de 20 et un maximum de 43. Le BMI moyen était de 28 ± 3 kg/m² dans le groupe « sortants » et de 29 ± 4 kg/m² dans le groupe « sorties ratées ». Les patients dans les deux groupes avaient des BMI comparables, donc nous n’avons pas étudié le BMI élevé comme facteur de risque de sorties ratées. Une majorité des patients ASA 1 (87% dans le groupe « sortants » et 82% dans le groupe « sorties ratées ») par rapport aux patients ASA 2 (13% dans le groupe « sortants » et 18% dans le groupe « sorties ratées ») a été retrouvée. La classe ASA 2 n’a pas été identifié comme facteur de risque de sorties ratées (p=0,4).

### Prémédication

Nous n’avons prescrit que les traitements habituels ne devant pas être arrêtés, sans aucune prémédication anxiolytique.

***Protocole anesthésique:*** Tous les patients ont bénéficié du même protocole anesthésique: 1) l’induction a été effectuée par du Propofol en anesthésie intra veineuse à objectif de concentration (AIVOC) avec une concentration cible cérébrale de 6 μg/ml selon le modèle de Schnider associé au Remifentanil en AIVOC avec une concentration cible cérébrale de 4ng/ml selon le modèle de Minto; 2) après l’intubation orotrachéale, nous avons administré en intraveineux (IV) :0,15 mg/kg de Kétamine (dose anti-hyperalgésique), 2g de Céfazoline comme antibio-prophylaxie et 8 mg de Déxaméthasone pour la prévention des nausées et vomissements postopératoires; 3) l’entretien de l’anesthésie a été fait par du Propofol en AIVOC avec une concentration cible cérébrale fixée à 2,5 μg/ml associé au Remifentanil en AIVOC avec une concentration cible cérébrale adaptée (+/- 0,5 ng/ml) à l’hémodynamique; 4) l’analgésie postopératoire a été anticipée en per opératoire par l’administration de 1g de Paracétamol, 100 mg de ketoprofen et 0,1 mg/kg de Morphine, 30 min avant la fin de l’intervention; 5) la sortie de la SSPI a été autorisée si le score d’Aldrete modifié a été égal à 10 sans douleurs post opératoires (DPO) (EVA « échelle visuelle analogique » < 30mm) ni NVPO. Dès la sortie de la SSPI, les patients ont eu le droit aux liquides per os autorisant un relais analgésique (Paracétamol 1g x 4/ jour) par la même voie. La durée de l’anesthésie était plus importante dans le groupe « sorties ratées » (76 ± 15 min) que dans le groupe « sortants » (67 ± 14 min).

La durée de l’anesthésie dépassait les 70 minutes chez 9 patients sur 11 du groupe « sorties ratées » et chez 9 patients sur 39 du groupe « sortants ». La durée de l’anesthésie > à 70 minutes a été retenue comme facteur de risque de sorties ratées avec une valeur de p à 0,001.

***Protocole chirurgicale***: Pour tous nos patients nous avons utilisé la technique classique dite à quatre trocarts. Nous avons procédé à une infiltration d’anesthésiques locaux au niveau du siège des orifices de trocart à la Bupivacaine isobare 0,125% (5cc/site d’incision), avant leurs mises en place. Nous avons limité la pression d’insufflation à 8 mm de Hg avec une augmentation progressive en cas de difficulté technique, sans dépasser 12mm de Hg. La durée moyenne de chirurgie était comparable entre les deux groupes avec 35 ± 12 min dans le groupe « sortants » et 42 ± 12 min dans le groupe « sorties ratées ». Une durée de la cholécystectomie supérieure à 40 minutes n’était pas un facteur de risque de sorties ratées (p=0.08). Tout nos patients ont été opérés par un chirurgien sénior afin d’assurer le maximum de sécurité et d’éviter ainsi les hospitalisations imprévues et les ré-hospitalisations pour complications chirurgicales. Nous résumons dans le Tableau 1 les résultats comparatifs des deux groupes sortants et sorties ratées.

**Tableau 1:** Résultats comparatifs des deux groupes, sortants et sorties ratées

		Groupe Sortants 39 patients 78%	Groupe Sorties ratées 11 patients 22%	p
**Age supérieur à 45 ans**		6	9	**0,000**
**Sexe masculin**		1	2	0,1
ASA	ASA 1	34	9	0,4
	ASA 2	5	2	
Durée de l’anesthésie supérieure à 70 minutes		9	9	**0,001**
Durée de l’intervention supérieure à 40 minutes		5	6	0,08

### Surveillance dans la SSPI

La durée du séjour dans la SSPI était plus longue pour les patients du groupe « sorties ratées » mais sans différence statistiquement significative entre les deux groupes (Tableau 2). Les EVA moyens de la DPO en SSPI et la consommation totale de Morphine étaient plus importants dans le groupe « sorties ratées » mais il n’y avait pas de différence significative entre les deux groupes (Tableau 2).

**Tableau 2:** Eléments de la surveillance dans la SSPI

	Sortants	Sorties ratées	p
SpO2 < 92% (Oui/non)	3/36	3/8	0,11
NVPO (Oui/non)	3/36	2/9	0,301
EVA moyen SSPI (cm)	1,73±1,91	2,55±1,57	0,213
Dose totale de Morphine (mg)	2,06±4,06	3,27±4,12	0,401
Durée du Séjour SSPI (min)	45,94 ±22,19	54,55 ±16,80	0,248

**Surveillance dans la salle d’hospitalisation (** **Tableau 3** **)**

**Tableau 3:** Eléments de la surveillance dans la salle d’hospitalisation

	Sortants	Sorties ratées	p
EVA moyen des DOP au service (cm)	1,21±1,065	1,56±0,924	0,336
NVPO (Oui/non)	1/38	1/10	0,395
DYSPHAGIE	1/38	0/11	0,78
PIC HYPERTENSIF	1/38	0/11	0.78
**FATIGUE (Oui/non)**	**0/39**	**4/7**	**0,01**
**CEPHALEES (Oui/non)**	**1/38**	**3/8**	**0,029**
**VERTIGES (Oui/non)**	**0/39**	**2/9**	**0,045**

Nous avons comparé les deux groupes concernant l’incidence des diverses plaintes. Dans le groupe « sortants » un patient s’est plaint d’une dysphagie (p=0,78), un deuxième a présenté un pic hypertensif (p=0,78). Par ailleurs nous avons constaté qu’il y avait une différence statistiquement significative entre les deux groupes concernant les autres plaintes: fatigue, céphalées et vertiges (Tableau 3). Avant la sortie, dans un entretien final avec chaque patient et son accompagnant, nous leurs avons expliqué les consignes postopératoires: 1) le traitement à prendre: Profenid 100mg x 2/j, Paracetamol 1 g x 4/j, Famotidine 1cp/j, Spasfon 2cp x 3/j et une prophylaxie thromboembolique pendant 5 jours; 2) les activités interdites et les précautions (conduite de véhicules, décision importante, utilisation d’engins dangereux); 3) l’alimentation: liquide puis semi-liquide le jour de l’opération, et retour progressif à une alimentation normale dès le lendemain; 4) les symptômes alertants (douleurs intenses, vomissements, vertiges) et le numéro de téléphone à appeler en cas de problèmes (disponible 24h/24h).

### Données après retour au domicile

**Figure 2:**
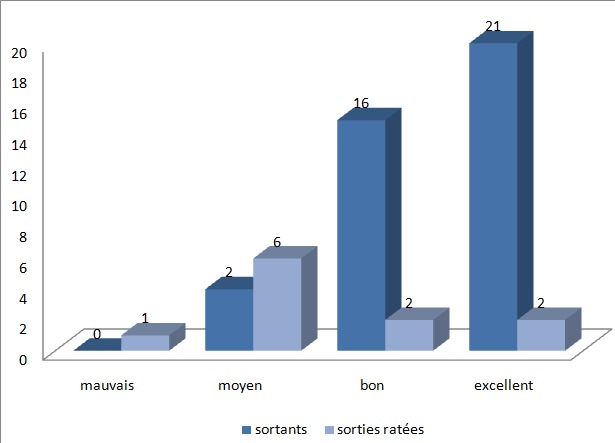
Taux de satisfaction des patients

Cinquante deux pour cent des patients ont rétabli leur transit le jour même de l’intervention (5/11 dans le groupe « sorties ratées » et 21/39 dans le groupe « sortants ») le reste des patients ont rétabli leur transit le lendemain. Seulement 5 patients du groupe « sortants » ont présenté des épisodes de NVPO après leur retour à domicile sans sentir le besoin de consulter ou de recevoir un traitement. Un seul patient du groupe « sorties ratées » a présenté des NVPO le lendemain de l’intervention. Concernant les DPO après le retour à domicile, il n’y avait pas de différence significative entre les EVA moyens des deux groupes à J0, J1 et J3. Deux patientes nous ont consultés pour des douleurs abdominales l’une à J5 post opératoire et l’autre à J9 post opératoire, toutes les deux se plaignaient d’une constipation. Aucun patient n’a été réadmis. Les patients qui ont pu être mis sortants ont été satisfaits du protocole de prise en charge avec des réponses majoritairement de type excellent et bon (94%) alors que les réponses dans le groupe « sorties ratées » étaient majoritairement de type mauvais et moyen (64%) (Figure 2).

## Discussion

### Controverse

La cholécystectomie laparoscopique (CL) est réservée essentiellement pour la prise en charge des calculs vésiculaires symptomatiques. Il existe une importante controverse quant au fait de savoir si elle devrait être pratiquée en chirurgie ambulatoire ou dans le cadre d'une chirurgie avec hospitalisation d'une nuit pour ce qui concerne la sécurité des patients. Nous pensons comme plusieurs autres auteurs que la CL s’apprête bien à une chirurgie ambulatoire du fait de sa courte durée, de la rareté des complications immédiates et de l’absence de perturbation du transit gastro-intestinal. Toutefois plusieurs séries rapportent des taux de sorties ratées supérieur à 20% [3, 4]. L’obstacle majeur au développement de la CLA est le dogme qu’ont plusieurs chirurgiens, quant au fait qu’un séjour d’au moins de 24 heures en postopératoire, est nécessaire pour détecter les complications postopératoires immédiates [5]. Toutefois, l’incidence des complications postopératoires majeures nécessitant une reprise chirurgicale est très basse (0,1-0,6% pour les plaies biliaires, et moins de 0,05% pour un saignement artériel). Aussi ces évènements sont détectés en per opératoire [6, 7], ou durant les 6 premières heures postopératoires et les autres complications comme les douleurs abdominales, la fièvre ou l’ictère ne se manifestent que quelques jours après la CLA [6, 7]. L’évaluation de l'impact de la cholécystectomie laparoscopique en chirurgie ambulatoire versus en chirurgie avec hospitalisation d'une nuit sur des critères de jugement axés sur le patient tels que : la mortalité, les graves événements indésirables et la qualité de vie, a fait l’objet de nombreux travaux. Malgré les résultats satisfaisants de ces travaux, la CLA n’est pas de pratique courante dans le monde, en effet elle présentait un taux de 11,3% au Royaume Uni en 2006, et selon les séries, ce taux variait de 37,9% à Singapour en 2006, 53% à Paris en 2005, 62% en Finlande en 2004 et jusqu’à 70% au Canada en 2003.

### Critères de sélection des patients

La sélection des patients candidats à une CLA passe par la définition des critères d’inclusion et des critères d’exclusion. Certains auteurs [8–10] pensent que ces critères sont trop stricts, ce qui explique le taux faible de la CLA par rapport à la cholécystectomie avec hospitalisation d’une nuit. Nous pensons après cette première expérience que la sensibilisation des patients et aussi de son entourage est primordiale pour augmenter le nombre de patients opérés en ambulatoire. L’étude comparative espagnole de Pérez a identifié trois types de critères d’inclusion [11]: 1) ***Critères liés à la pathologie:*** L’absence d’hospitalisation pour pancréatite aigue ou cholécystite aigue au cours des trois derniers mois, l’absence de lithiase de la voie biliaire principale, et un bilan hépatique non perturbé; 2) ***Critères généraux:*** L’absence de chirurgie supra-mésocolique majeure, pas de prise d’anticoagulants ou d’anti agrégants plaquettaires, une classe ASA 1 et 2; 3) ***Critères sociaux:*** La distance de l’hôpital, le soutien familial.

Dans la Cohorte espagnole de 1600 CLA, la Bili-IRM préopératoire, trouve sa place chez les patients présentant des facteurs prédictifs de lithiase de la voie biliaire principale [10] et cela après la réalisation systématique d’un bilan hépatique et d’une échographie abdominale. Les critères d’exclusion qui reviennent le plus souvent dans la littérature [8], sont: 1) les malades ASA 3 et 4; 2) les grands obèses; 3) les malades de plus de 70 ans; 4) les malades aux antécédents de chirurgie abdominale; 5) les malades suspects de lithiase de la voie biliaire principale, de pancréatite aigue ou de cholécystite aigue. Dans la série Italienne de 2012, les antécédents de chirurgie abdominale (même de l’étage sus-mésocolique) n’étaient pas un critère d’exclusion formel [9].

### Organisation de l’unité de chirurgie ambulatoire

Tous les auteurs s’accordent pour commencer la première CLA dans la journée, tôt le matin. Ceci permettrait d’éviter au maximum les sorties ratées [8, 10]. Onze heures du matin a été fixée arbitrairement par la plupart des séries, comme heure limite de démarrage de la dernière CLA de la journée [9] pour pouvoir garder le patient au moins 6 heures en post opératoire, sachant que les sorties se font au plus tard vers 19 heures.

### Protocole anesthésique

Nous avons utilisé le Propofol à l'induction et à l'entretien, à la fois pour ses propriétés pharmacocinétiques, et parce qu'il prévient les nausées et vomissements post opératoires. En effet, une méta-analyse [12] a comparé le Propofol aux différents halogénés concernant la récupération après anesthésie. Le retour au domicile était plus rapide avec le Propofol et le Desflurane par rapport au Sevoflurane et à l’Isoflurane. Les céphalées, les nausées et vomissements en post opératoire immédiat et à 24h étaient plus marqués avec les halogénés qu’avec le Propofol. La nécessité de traiter ces nausées et vomissements était plus importante avec le Sevoflurane et le Desflurane [12]. Il a été démontré que l’anesthésie intraveineuse au Propofol réduit l’incidence et la sévérité des NVPO avec moins 30% de risque de survenue de cette complication [12–16]. Ceci permet une meilleure qualité de réveil, un transfert direct de la salle d’opération au service d’hospitalisation (fast-tracking) et une plus grande satisfaction des patients [17–19]. Nous avons choisi le Remifentanil comme opioïde pour ses propriétés pharmacocinétiques favorables à l’anesthésie ambulatoire avec sa bonne maniabilité per opératoire et la possibilité d'un réveil quasi immédiat (court délai d’action et courte demi-vie d’élimination: 3-5 min). Malgré ses avantages théoriques, le Remifentanil ne semble pas modifier la durée du séjour hospitalier. En anesthésie ambulatoire, lorsqu’une curarisation prolongée est nécessaire on utilise des curares non dépolarisants. Or La curarisation pour une cholécystectomie laparoscopique est généralement nécessaire et le choix de la molécule varie selon la durée attendue du geste [20–22]. Bien que le Mivacurium (Mivacron) soit le curare de choix en anesthésie ambulatoire permettant souvent d’éviter l’antagonisation [23], nous avons opté pour l’utilisation du cisatracurium.

En effet, il s’agit d’un curare non dépolarisant, qui par sa durée d’action intermédiaire et moyennant une injection unique peut couvrir toute la période opératoire sans recours à une injection complémentaire. Ceci a été validé par notre étude, seulement deux patients sur les 50 inclus non exclus ont nécessité un bolus complémentaire. Au décours d’une cholécystectomie laparoscopique ambulatoire, la Déxaméthasone améliore la récupération post opératoire intra hospitalière et même après le retour a domicile [24]. En effet, elle réduit significativement l’incidence des NVPO, des douleurs, de la consommation analgésique, de la fatigue et de la durée de convalescence avec un meilleur état émotionnel et un meilleur confort physique [24–29]. La dose minimale recommandée est de 8 mg [30, 31] même si des doses inférieures se sont avérées efficaces [32–34]. A cette dose aucun effet indésirable n’a été signalé. Wang et al ont montré que l’administration de la Déxaméthasone avant l’induction était plus efficace que quand elle a été administrée à la fin de l’opération [35]. Comme dans la série anglaise de 2009 [36], nous avons administré ce corticoïde immédiatement après l’induction pour éviter les fourmillements périnéaux qui pourraient accompagner son injection [37]. Le metoclopramide a été utilisé en fin d’intervention, pour la prévention des NVPO, et ceci dans plusieurs séries dont l’essai clinique randomisé de Johansson [8]. Des études ont montré que le protoxyde d’azote (N2O) associé au Propofol pour l’anesthésie ambulatoire n’affecte ni le délai de sortie à domicile ni l’incidence des nausées et vomissements [38]. Les résultats de plusieurs études justifient que l’éviction du N2O fait partie des stratégies multimodales de prévention des NVPO, dans le cadre de l’approche ambulatoire surtout chez les patients à risque [39–41]. Pour cette raison, nous n’avons pas utilisé ce gaz anesthésique. En fin d’intervention et avant l’extubation, nous avons procédé à une aspiration complète du contenu de l’estomac pour assurer une décompression gastrique. Ceci pourrait réduire l’incidence et la sévérité des NVPO [14].

La physiopathologie de la douleur après cholécystectomie laparoscopique est complexe et résulte essentiellement de: 1) douleurs au niveau des sites d’incision; 2) distension péritonéale secondaire au volume résiduel du C02 intra péritonéal; 3) traumatisme local du lit de dissection vésiculaire; 4) la péritonite chimique secondaire à l’extravasation péritonéale de la bile [14]. Sur cette base physiopathologique nous avons choisi d’associer plusieurs thérapeutiques afin d’assurer une analgésie préventive et multimodale. L’analgésie multimodale a pour but d’associer différentes molécules ayant un mécanisme d’action différent dans l’espoir de renforcer l’analgésie postopératoire et/ou diminuer les besoins en analgésiques et leurs effets secondaires. Ces objectifs sont principalement atteints lors de l’utilisation de morphiniques [42].

Notre protocole analgésique regroupe: 1) l’infiltration des sites d’incision par les anesthésiques locaux; 2) la kétamine; 3) les anti-inflammatoires non stéroïdiens; 4) le paracétamol; 5) les opioïdes. Ce type d’approche a montré son efficacité, offrant un contrôle presque total de la douleur pendant la période postopératoire. Cette analgésie pourrait participer à une récupération rapide, un séjour limité en SSPI et ainsi au succès de la prise en charge ambulatoire de la CL [14, 43].

### Protocole chirurgical

Le choix de l’opérateur est un facteur primordial dans la chirurgie ambulatoire d’une façon générale et particulièrement dans la cholécystectomie laparoscopique: d’une part pour la sécurité du patient, surtout qu’il devrait quitter l’hôpital le jour même, pour pouvoir conclure à la sûreté de la CLA et d’autre part pour raccourcir le temps opératoire dont l’allongement serait un des facteurs de sorties ratées selon certains auteurs [44]. Dans la littérature, les CLA on étés réalisées soit par un chirurgien sénior, soit par un résident avec l’aide d’un chirurgien expérimenté [8, 9]. Pour Pérez, il est primordial que la CLA soit faite par un chirurgien habitué. En effet, il doit avoir effectué au moins 50 cholécystectomies par voie coelioscopique avant d’opérer en ambulatoire [11].

**Nombre de trocarts:** Toutes les séries décrivent une technique à quatre trocarts, adoptée aussi par notre équipe, à l’exception des Italiens [9] qui n’utilisent que trois trocarts. Ils se passant ainsi d’un trocart de 10mm au niveau du flanc gauche pour minimiser au maximum les douleurs post opératoires.

**Pression d’insufflation:** Comme pour le nombre de trocarts, Brescia [9] se démarque par l’utilisation d’une faible pression d’insufflation de CO2 entre 8 et 9 mmHg.

**Cholangiographie per opératoire:** Elle est de pratique systématique dans l’essai de Johansson [8]. Dans la cohorte de 1600 cas, elle est faite lorsqu’il y a un doute sur la bili-IRM [10]. Nous n’avons réalisé aucune Cholangiographie per-opératoire au cours de notre étude, les patients qui avaient des signes prédictifs de lithiase de la voie biliaire associée étant exclus d’emblée.

**Drain de Redon:** Son utilisation est réduite au minimum [9, 10]. Tous les auteurs insistent sur la rigueur dans la technique de la cholécystectomie qui doit respecter les règles de l’hémostase et de la bilistase décrite par Strasberg [45] et Rouviere [46] évitant ainsi le drainage qui selon Akoh [44] est un facteur « de sortie ratée ».

Trois essais ont étudié l’effet de la mise en place d’un drainage du site opératoire. Les deux premiers [47, 48] ont placé des drains en sous hépatique, et ils ont noté une élévation significative des douleurs post opératoires comparativement aux patients qui n’ont pas eu de drainage. Dans le troisième essai, Nursal [49] a placé des drains en inter hépato diaphragmatique dans le but d’évacuer le gaz résiduel suivant l’insufflation, et il n’y a pas eu de différence significative pour l’utilisation des antalgiques et des anti-émétiques comparativement au groupe qui n’a pas eu de drainage.

### Surveillance dans la SSPI

Après leur réveil, les patients sont transférés dans une unité de prise en charge dédiée à la chirurgie ambulatoire, où le premier lever et un régime liquide sont conseillés dès que possible [8, 10]. L’étude Italienne, portant sur 400 CLA, a fixé 14 heures comme l’heure de reprise du régime liquide [9]. Une analgésie à base de diclofène à la dose de 50mg trois fois par jour et de paracétamol à la dose de 1g quatre fois par jour le premier jour est de mise pour certaines équipes [8]. Brescia [9], dans sa série, préconise de réaliser systématiquement une NFS à 16 heures, vu que dans son étude le drainage n’a pas été utilisé (pour diminuer les douleurs post opératoires). Enfin les patients sont toujours examinés par un chirurgien avant leurs sorties. Dans la Cohorte espagnole de 1600 cas [10], cette visite peut avoir lieu dans un intervalle allant de 16 heures à 19 heures. Brescia [9], quant à lui, a choisi 18 heures comme l’horaire de la visite avant la sortie du malade, et il a aussi exigé la présence du médecin anesthésiste au cours de celle-ci.

### Faisabilité de la CLA

Trois essais cliniques randomisés ont comparés la CLA à la cholécystectomie avec une hospitalisation d’une nuit [50–52], et ils ont démontré la faisabilité de la CLA. Dans l’essai clinique randomisé de Johansson [8], un taux de succès de 92% a été observé. L’étude Italienne de 2013, portant sur 400 patients [9] a eu un taux de succès de 96,7%. Ceci est en majorité la conséquence des critères de sélection choisis. En effet, dans cette étude l’âge moyen des patients était de 52 ans et le BMI moyen égal à 29,3Kg/m². On note ainsi une amélioration des taux de succès avec le temps, comme vient le confirmer la cohorte espagnole de 1600 CLA consécutives s’étalant le 1997 à 2010 [10]. De tels progrès sont le fruit de la compréhension des mécanismes physiopathologiques de la douleur, permettant ainsi un meilleur contrôle de celle-ci, et de l’utilisation de drogues anesthésiques compatibles avec une chirurgie en ambulatoire.

### Sorties ratées

Nous résumons dans le Tableau 4, le taux de sorties ratées dans les différentes séries de la littérature. Le taux de sorties ratées élevé dans certaines séries [51–54] peut être expliqué par la mauvaise gestion des douleurs et nausées postopératoires, comme dans l’expérience de Dirksen [51] et Hollington [52]. L’instauration de critères de sélection non stricts peut aussi expliquer le taux élevé de sorties ratées, comme en témoigne l’étude de Akoh [44], où des patients ASA 3, aux antécédents de pancréatite aigue ou de cholécystite aigue ont étés inclus dans l’étude. Ceci aura pour conséquence la majoration des difficultés per-opératoires, l’allongement de la durée opératoire et l’augmentation des doses de drogues anesthésiques.

**Tableau 4:** Sorties ratées

Auteurs	Année	Série	Nb CLA/CLL	Sorties ratées CLA
Keulemans.Y [50]	1998	Essai clinique randomisé	37/43	8%
Hollington.P [52]	1999	Prospective randomisée	60/71	18%
Dirksen.CD [51]	2001	Randomisée	42/44	26%
Johansson.M [8]	2005	Essai clinique randomisé	52/48	8%
Akoh.A [44]	2011	Rétrospective	258/0	31%
Ahn.Y [53]	2011	Revue systémique	/	20%
Brescia.A [9]	2013	Rétrospective	400/0	3.3%
Roig.MP [10]	2013	Prospective contrôlée	1600/0	4.6%
Vaughan.J [54]	2013	Cochrane Data base syst rev	205/214	19.3%
Perez.MA [11]	2013	Rétrospective	141/286	18%

Nb CLA/CLL: nombre de cholécystectomie laparoscopique ambulatoire/et avec une hospitalisation d’une nuit

### Raisons et facteurs prédictifs de sorties ratées

Une première série française [55] a analysé via une étude statistique uni variée les facteurs prédictifs de sorties ratées après une CLA sur une série de 217 malades. Les auteurs de ce travail ont conclu que l’âge supérieur à 65 ans, l’allongement de la durée opératoire et le début tardif de la chirurgie après 11 heures du matin sont les seuls facteurs responsables de sorties ratées. En vue d’améliorer les résultats de la CLA, l’identification des raisons de sorties ratées est primordiale. En effet, dans la cohorte espagnole de 1600 CLA [10], une analyse multi variée a été réalisée, étudiant ainsi l’âge des patients, la durée de l’intervention, le sexe, la durée entre le réveil du malade et sa sortie, l’horaire de la chirurgie (matin/après midi) et la chirurgie à l’hôpital ou en ville. Il s’est avéré que seul l’âge des patients et l’horaire de la chirurgie sont des facteurs prédictifs de sorties ratées. Dans l’étude anglaise de 2011 [44], la durée de l’intervention, la classe ASA et le sexe ont été étudiés, et aucun facteur prédictif n’a été individualisé. Cependant, Robinson, dans son étude portant sur 289 CLA [56], a objectivé que la tranche d’âge entre 50 à 65 ans est un facteur prédictif de sorties ratées. Dans une analyse rétrospective colligeant 731 cas de CLA, Lau et Brooks [57] ont identifié la durée de l’intervention comme le meilleur facteur prédictif de sorties ratées. Une étude espagnole récente, publiée en 2013 [11], a un taux de sortie ratées de 18%, en rapport avec un mauvais contrôle des douleurs postopératoires, des difficultés opératoires, des NVPO, l’instabilité de l’état hémodynamique, la rétention urinaire, des raisons sociales, et la conversion en laparotomie. Dans notre série l’âge supérieur à 45 ans et la durée de l’anesthésie supérieure à 70 minutes ont été individualisés comme facteurs prédictifs de sorties ratées.

**Réadmissions:** Dans notre série, aucun patient n’a été réadmis. Dans la littérature le taux de réadmission diffère selon les auteurs, comme illustré dans le Tableau 5).

**Tableau 5:** Taux de réadmissions après CLA

Auteurs	Année	Série	Nb CLA/CLL	% réadmission CLA
Kavanagh.T [58]	2008	Prospective	40/0	12.5%
Akoh.JA [44]	2011	Rétrospective	258/0	5.2%
Johansson.M [8]	2005	Essai clinique randomisé	52/48	0%
Roig.MP [10]	2013	Prospective contrôlée	1600/0	2.1%
Vanghan.J [54]	2013	Cochrane Database syst rev	136/154	3.5%

Nb CLA/CLL: nombre de cholécystectomie laparoscopique ambulatoire/et avec une hospitalisation d’une nuit

**Causes de réadmissions:** Les réadmissions sont dues pour la plupart à un mauvais contrôle des douleurs postopératoires comme a conclu Akoh dans sa série rétrospective [44]. Hormis les douleurs postopératoires, Kavanagh a pu isoler d’autres causes de réadmission qui sont les nausées et vomissements postopératoires [58].

**Sûreté:** Dans la Cohorte espagnole de 1600 CLA, 10 d’entres elles ont été grevées d’une collection sous hépatique postopératoire, dont la cause était une fistule biliaire par un canal de Luschka chez 3 patients, et une origine non biliaire chez trois autres [10]. Briggs a rapporté un seul cas de complication postopératoire, il s’agissait d’une collection sous hépatique inaccessible à un drainage percutané, pour laquelle le patient a été repris chirurgicalement et a eu une toilette péritonéale avec mise en place d’un drainage (l’origine de la fistule biliaire n’a pas été individualisé) [36]. Dans l’étude de Perez, deux patients ont été réopérés après que le diagnostic d’hémopéritoine ait été porté [11]. Malgré la présence d’une morbidité après une CLA, tous les auteurs s’accordent à dire qu’il n’existe pas de différence significative concernant la morbidité entre le groupe CLA et le groupe cholécystectomie avec hospitalisation d’une nuit. En effet, la plupart des complications sont diagnostiquées durant l’acte chirurgical ou après 48 heures de celui-ci [10, 11, 53, 59, 60]. La majorité des séries, y compris la notre, ne rapporte aucun décès dans les suites d’une CLA. Un unique décès a été rapporté par Roig, dû à une hernie de Richter à travers l’orifice de trocart ombilical [10]. La satisfaction des patients est un fait fondamental à prendre en considération dans la chirurgie en ambulatoire, notamment la CLA. Dans des séries précédentes, le taux de satisfaction variait de 60 à 95% [50, 53, 61–63]. Dans notre série, les patients qui ont pu être mis sortants ont été satisfaits du protocole de prise en charge avec des réponses majoritairement de type excellent et bon (94%). Un tel écart dans les taux de satisfaction peut être en corrélation directe avec les instruments de mesure de ce paramètre. Ceux-ci doivent être sensibles, cohérents, reproductibles, applicables et validés, permettant d’estimer la satisfaction du patient sur des critères objectifs et non sur une impression générale [8].

### Intérêts économiques de la CLA

Comme nous l’avons souligné précédemment, la CLA et la CL avec hospitalisation d’une nuit, se valent en termes de morbidité et de mortalité. Dans notre étude nous n’avons pas comparé le coût des deux procédures, et pour faire ressortir l’intérêt économique de la CLA nous avons effectué une revue de la littérature. Johansson [8], dans son essai clinique randomisé a montré que le coût moyen de la CLA était moins élevé que celui d’une CL avec hospitalisation d’une nuit (3085 € Vs 3394 €). En terme économique, la CLA diminue les coûts de 11% par rapport à l’autre procédure et ceci passe par la réduction des dépenses post opératoires, dont le nursing représente 31% [64]. En 2009 en Espagne, 31131 CL [65] ont été réalisées, avec un séjour hospitalier s’étendant de 2.1 à 3.5 jours. Le recours à la CLA aurait fait économiser 18 millions d’Euros [65].

### Comment améliorer les résultats

Suite à notre étude et à une revue de la littérature, nous avons pu individualiser des mesures permettant d’améliorer les résultats de la CLA. Ces mesures ont pour but de diminuer les douleurs, les nausées et vomissements postopératoires qui comme on l’a souligné plus haut, sont les principales causes de sorties ratées et de réadmissions. On peut diviser ces mesures en trois groupes : mesures préopératoires, mesures per opératoires et mesures post opératoires. Les mesures préopératoires sont la correction de la déshydratation préopératoire (niveau de recommandations IB) [3], l’administration de la Déxaméthasone 8 mg en IV (niveau de recommandations IA) qui devrait être 90 min avant l’induction [28, 66], l’administration d’anti inflammatoires type AINS ou d’inhibiteur de la COX II (niveau de recommandations IA) [53] et enfin l’éducation des patients (niveau de recommandations IIB) [67, 68]. Les mesures per opératoires sont l’administration d’apport classique de fluides (niveau de recommandations IB) [69], l’administration de sulfate de magnésium en perfusion (niveau de recommandations IB) [70], l’utilisation d’une pression de pneumopéritoine inférieure à 9mmHg de CO2 (niveau de recommandations IB) [53], une anesthésie locale pré incisionnelle des plaies opératoires et du péritoine (niveau de recommandations IA) [71, 72] et la prescription d’anti émétiques (niveau de recommandations IA) [73, 74]. Le drainage du site opératoire ne doit pas être fait de manière systématique (niveau de recommandations IA).

## Conclusion

Nous concluons après cette première expérience que la chirurgie ambulatoire est tout aussi sûre que la chirurgie avec hospitalisation d'une nuit dans la cholécystectomie laparoscopique. Notre taux de prise en charge en ambulatoire est significativement plus faible que celui des séries de la littérature et est inférieur à celui que nous avions prévu initialement. Une forte sensibilisation des patients et des médecins aurait pu permettre d’éviter les cas où aucun facteur d’exclusion n’a pu être trouvé. Les résultats des différents travaux publiés suggèrent que cette chirurgie ambulatoire est sûre pour les patients. Il est important de remarquer que tous les essais présentaient un risque de biais et que les données étaient rares, ce qui a donné un risque considérable de parvenir à de mauvaises conclusions en raison d'erreurs systématiques (surestimation des bénéfices ou sous-estimation des préjudices de la chirurgie ambulatoire ou du séjour d'une nuit à l'hôpital) et d'erreurs aléatoires (effet du hasard). D'autres essais randomisés doivent être réalisés pour étudier l'impact de la chirurgie ambulatoire et du séjour d'une nuit à l'hôpital sur la qualité de vie et d'autres critères de jugement pour les personnes subissant une cholécystectomie laparoscopique.

### Etat des connaissances actuelles sur le sujet

- Les études provenant d’occident concluent que la chirurgie en ambulatoire pour lithiase vésiculaire simple, est aujourd'hui faisable et sûre avec un gain économique considérable. Toutefois ces publications sont hétérogènes et la grande majorité provient de l’Europe ou des Etas unis, posant le problème de la transposabilité des résultats sous nos cieux.

### Contribution de notre étude à la connaissance

- La chirurgie ambulatoire est tout aussi sûre que la chirurgie avec hospitalisation d'une nuit dans la cholécystectomie laparoscopique en respectant des critères de sélections strictes;
- Un nombre important de patients aurait pu bénéficier d’une prise en charge en ambulatoire. Une forte sensibilisation des patients et des médecins aurait pu permettre d’éviter les cas où aucun facteur d’exclusion n’a pu être trouvé.

## Conflits d’intérêts

Les auteurs ne déclarent aucun conflit d'intérêts.

## References

[1] Kraft K, Mariette C, Sauvanet A (2011). Indications for ambulatory gastrointestinal and endocrine surgery in adults. J Visc Surg..

[2] Nordin P, Haapaniemi S, van Der Linden W, Nilsson E (2004). Choice of anesthesia and risk of reoperation for recurrence in groin hernia repair. Ann Surg..

[3] Adanir T, Aksun M, Ozgurbuz U, Altin F, Sencan A (2008). Does preoperative hydration affect postoperative nausea and vomiting? A randomized, controlled trial. J Laparoendosc Adv Surg Tech A..

[4] Hausel J, Nygren J, Thorell A, Lagerkranser M, Ljungqvist O (2005). Randomized clinical trial of the effects of oral preoperative carbohydrates on postoperative nausea and vomiting after laparoscopic cholecystectomy. Br J Surg..

[5] Saunders CJ, Leary BF, Wolfe BM (1995). Is outpatient laparoscopic cholecystectomy wise? Surg Endosc.

[6] Gentileschi P, Di Paola M, Catarci M, Santoro E, Montemurro L, Carlini M (2004). Bile duct injuries during laparoscopic cholecystectomy. Surg Endosc..

[7] Connor S, Garden OJJ (2006). Bile duct injury in the era of laparoscopic cholecystectomy. Br J Surg..

[8] Johansson M, Thune A, Nelvin L (2006). Randomized clinical trial of day-care versus overnight-stay laparoscopic cholecystectomy. Br J Surg..

[9] Antonio Brescia, Marcello Gasparrini, Giuseppe Nigri, Umile Michele Cosenza, Anna Dall’Oglio, Alessandra Pancaldi (2013). Laparoscopic cholecystectomy in day surgery: Feasibility and outcomes of the first 400 patients. The surgeon..

[10] Manuel Planells Roig, Rafael Garcia Espinosa, Maria Cervera Delgado, Francisco Navarro Vicente, Miguel Carrau Giner, Angel Sanahuja Santafe (2013). Ambulatory laparoscopic cholecystectomy - a cohort study of 1600 consecutive cases. Cir esp..

[11] Maria Angeles Lezana Pérez, Guillermo Carreno Villarreal, Paola Lora Cumplido, Raul Alvarez Obregon (2013). Comparative study of ambulatory laparoscopic cholecystectomy versus management of laparoscopic cholecystectomy with conventional hospital stay. Cir esp..

[12] Gupta A, Stierer T, Zuckerman R (2004). Comparison of recovery profile after ambulatory anesthesia with Propofol, Isoflurane, Sevoflurane and Desflurane: a systematic review. Anesth Analg..

[13] Purhonen S, Koski E, Niskanen M (2006). Efficacy and costs of 3 anesthetic regimens in the prevention of postoperative nausea and vomiting. J Clin Anesth..

[14] Michaloliakou C, Chung F, Sharma S (1996). Preoperative multimodal analgesia facilitates recovery after ambulatory laparoscopic cholecystectomy. Anesth Analg..

[15] Tramer M, Moore A, McQuay H (1997). Propofol anaesthesia and postoperative nausea and vomiting: quantitative systematic review of randomized controlled studies. Br J Anaesth..

[16] Apfel C, Roewer N (2004). Postoperative nausea and vomiting. Anaesthesist..

[17] Tang J, Chen L, White P (1999). Recovery profile, costs and patient satisfaction for fast-track office-based anesthesia. Anesthesiology..

[18] Eberhart L, Eberspaecher M, Wulf H (2004). Fast-track eligibility, costs and quality of recovery after intravenous anaesthesia with propofol-remifentanil versus balanced anaesthesia with isoflurane-alfentanil. Eur J Anaesth..

[19] Elliott R, Payne K, Moore J (2003). Clinical and economic choices in anaesthesia for day surgery: prospective randomised controlled trial. Anaesthesia..

[20] Damen S, Nieuwenhuijs V, Joosten W (2004). The effects of remifentanil and sufentanil on the quality of recovery after day case laparoscopic cholecystectomy: a randomized blinded trial. Journal of Laparoendoscopic & Advanced Surgical Techniques..

[21] Prabhu A, Chung F (2001). Anaesthetic strategies towards developments in day care surgery. Eur J Anaesthesiol..

[22] Bazin J, Waleckx P, Slim K (2006). Spécificités de l'anesthésie en chirurgie abdominale de l'adulte par laparoscopie. EMC Anesthésie-Réanimation.

[23] Joshi G, Garg S, Hailey A (1999). The effects of antagonizing residual neuromuscular blockade by neostigmine and glycopyrrolate on nausea and vomiting after ambulatory surgery. Anesth Analg..

[24] Murphy G (2011). Preoperative dexamethasone. Anesthesiology..

[25] Fukami Y, Terasaki M, Okamoto Y et Al (2009). Efficacy of preoperative dexamethasone in patients with laparoscopic cholecystectomy: a prospective randomized double-blind study. J Hepatobiliary Pancreat Surg..

[26] Huang J, Shieh J, Tang C (2001). Low-dose dexamethasone effectively prevents postoperative nausea and vomiting after ambulatory laparoscopic surgery. Can J Anaesth..

[27] Wang J, Ho S, Liu Y (1999). Dexamethasone reduces nausea and vomiting after laparoscopic cholecystectomy. Br J Anaesth..

[28] Bisgaard T, Klarskov B, Kehlet H, Rosenberg J (2003). Preoperative dexamethasone improves surgical outcome after laparoscopic cholecystectomy: a randomized double-blind placebo-controlled trial. Ann Surg..

[29] Zargar-Shoshtari K, Sammour T, Kahokehr A (2009). Randomized clinical trial of the effect of glucocorticoids on peritoneal inflammation and postoperative recovery after colectomy. Br J Surg..

[30] Elhakim M, Nafie M, Mahmoud K (2002). Dexamethasone 8 mg in combination with ondansetron 4 mg appears to be the optimal dose for the prevention of nausea and vomiting after laparoscopic cholecystectomy. Can J Anaesth..

[31] Lee Y, Lai HY, Lin PC (2004). A dose ranging study of dexamethasone for preventing patient-controlled analgesia-related nausea and vomiting: a comparison of droperidol with saline. Anesth Analg..

[32] Coloma M, White P, Markowitz S (2002). Dexamethasone in combination with dolasetron for prophylaxis in the ambulatory setting: effect on outcome after laparoscopic cholecystectomy. Anesthesiology..

[33] Eberhart L, Mauch M, Morin A (2002). Impact of a multimodal anti-emetic prophylaxis on patient satisfaction in high-risk patients for postoperative nausea and vomiting. Anaesthesia..

[34] Wang J, Ho S, Uen Y (2002). Small-dose dexamethasone reduces nausea and vomiting after laparoscopic cholecystectomy: a comparison of tropisetron with saline. Anesth Analg..

[35] Wang JJ, Ho ST, Tzeng JI, Tang CS (2000). The effect of timing of dexamethasone administration on its efficacy as a prophylactic antiemeticfor postoperative nausea and vomiting. Anesth Analg..

[36] Briggs CD, Irvin GB, Mann CD, Cresswell A, Englert L, Peterson M (2009). Introduction of a day-case laparoscopic cholecystectomy service in the UK: a critical analysis of factors influencing same-day discharge and contact with primary care providers. Ann R Coll Surg Engl..

[37] Neff S, Stapelberg E, Warmington A (2002). Excruciating perineal pain after intravenous dexamethasone. Anaesth Intensive Care..

[38] Arellano R, Pole M, Rafuse S (2000). Omission of nitrous oxide from a propofol-based anesthetic does not affect the recovery of women undergoing outpatient gynecologic surgery. Anesthesiology..

[39] Felts J, Poler S, Spitznagel E (1990). Nitrous oxide, nausea, and vomiting after outpatient gynecologic surgery. J Clin Anesth..

[40] Mraovic B, Simurina T, Sonicki Z (2008). The dose-response of nitrous oxide in postoperative nausea in patients undergoing gynecologic laparoscopic surgery: a preliminary study. Anesth Analg..

[41] Ewart L (2010). Nitrous oxide makes me sick: or does it? Nitrous oxide and postoperative nausea and vomiting. J Perioper Pract..

[42] Comité douleur-anesthésie locorégionale et le comité des référentiels de la Sfar (2008). Recommandations formalisées d’experts 2008 - Prise en charge de la douleur postopératoire chez l’adulte et l’enfant. Ann Fr Anesth Reanim..

[43] Langloÿs J, Kamran S (2003). Anesthesie Ambulatoire. Anesthésie Réanimation Chirurgicale.

[44] Jacob Akoh A, Will Watson A, Thomas Bourne P (2011). Day case laparoscopic cholecystectomy: reducing the admission rate. International Journal of Surgery..

[45] Strasberg S, Hertl M, Soper N (1995). An analysis of the problem of biliary injury during laparoscopic cholecystectomy. J Am Coll Surg..

[46] Hugh TB, Kelly MD, Meisick A (1997). Rouviere's sulcus: a useful landmark in laparoscopic cholecystectomy. Br J Surg..

[47] Uchiyama K, Tani M, Kawai M, Terasawa H, Hama T, Yamaue H (2007). Clinical significance of drainage tube insertion in laparoscopic cholecystectomy: a prospective randomized controlled trial. J Hepatobiliary Pancreat Surg..

[48] Tzovaras G, Liakou P, Fafoulakis F, Baloyiannis I, Zacharoulis D, Hatzitheofilou C (2009). Is there a role for drain use in elective laparoscopic cholecystectomy? A controlled randomized trial. Am J Surg..

[49] Nursal TZ, Yildirim S, Tarim A, Noyan T, Poyraz P, Tuna N (2003). Effect of drainage on postoperative nausea, vomiting, and pain after laparoscopic cholecystectomy. Langenbecks Arch Surg..

[50] Kleusmans Y, Eshuis J, de Haes H, de Wit LT, Gouma DJ (1998). Laparoscopic chlecystectomy:day-care versus clinical observation. Ann surg..

[51] Driksen CD, Schmitz RF, Hans KM, Nieman FH, Hoogenboom LJ, Go PM (2001). Ambulatory laparoscopic cholecystectomy is as effective as hospitalization and from a social perspective less experience: a randomized study. Ned Tijdscbr Geneeskd..

[52] Hollington P, Toogood Gj, Padbury RT (1999). A prospective randomized trial of day-stay only versus overnignt stay laparoscopic cholecystectomy. Aust NZF Surg..

[53] Ahn Y, Woods J, Connor S (2011). A systematic review of interventions to facilitate ambulatory laparoscopic cholecystectomy. HPB (Oxford)..

[54] Vaughan J, Gurusamy KS, Davidson BR (2013). Day-surgery versus overnight stay surgery for laparoscopic cholecystectomy. Cochrane Database Syst Rev..

[55] Vandenbroucke F, Létourneau R, Roy A, Dagenais M, Bellemare S, Plasse M, Lapointe R (2007). Cholécystectomie coelioscopique ambulatoire: expérience d’un an sur des patients non sélectionnés. JCHIR..

[56] Robinson TN, Biffl WL, Moore EE, Heimbach JK, Calkins CM, Burch JM (2002). Predicting failure of outpatient laparoscopic cholecystectomy. Am J Surg..

[57] Lau H, Brooks DC (2001). Predictive factors for unanticipated admissions after ambulatory laparoscopic cholecystectomy. Arch Surg..

[58] Kavanagh T, Hu P, Minogue S (2008). Daycase laparoscopic cholecystectomy: a prospective study of post-discharge pain, analgesic and antiemetic requirements. Ir J Med Sci..

[59] Martinez Rodenas F, Hernandez Borlan R, Guerrero de la Rosa Y, Moreno Solorzano J, Alcaide Garriga A, PouSanchis E (2008). Colecistectomia laparoscopica ambulatoria: resultados iniciales de una serie de 200 casos. Cir Esp..

[60] Gurusamy K, Junnarkar S, Farouk M, Davidson BR (2008). Metaanalysis of randomised controlled trials on the safety and effectiveness of day-case laparoscopic cholecystectomy. Br J Surg..

[61] Mjaland O, Raeder J, Aasboe V, Trondsen E, Buanes T (1997). Outpatient laparoscopic cholecystectomy. Br J Surg..

[62] Richardson WS, Fuhrman GS, Burch E, Bolton JS, Bowen JC (2001). Outpatient laparoscopic cholecystectomy - Outcomes of 847 planned procedures. Surg Endosc..

[63] Siu WT, Leong HT, Law BK, Onsiong SM, Fung KH, Li AC (2001). Outpatient laparoscopic cholecystectomy in Hong Kong: patient acceptance. Surg Laparosc Endosc Percutan Tech..

[64] Rosen MJ, Malm JA, Tarnoff M, Zuccala K, Ponsky JL (2001). Cost effectiveness of ambulatory laparoscopic cholecystectomy. Surg Laparosc Endosc Percutan Tech..

[65] (2011). Estadisticas de Establecimientos Sanitarios con regimen de internado 2009.

[66] Fujii Y, Saitoh Y, Tanaka H, Toyooka H (2000). Granisetron/dexamethasone combination for the prevention of postoperative nausea and vomiting after laparoscopic cholecystectomy. Eur J Anaesthesiol..

[67] Blay N, Donoghue J (2006). Source and content of health information for patients undergoing laparoscopic cholecystectomy. Int J Nurs Pract..

[68] Stergiopoulou A, Birbas K, Katostaras T, Diomidous M, Mantas J (2006). The effect of a multimedia health educational programme on the postoperative recovery of patients undergoing laparoscopic cholecystectomy. Stud Health Technol Inform..

[69] Holte K, Klarskov B, Christensen D, Lund C, Nielsen K, Bie P (2004). Liberal versus restrictive fluid administration to improve recovery after laparoscopic cholecystectomy. Ann Surg..

[70] Mentes O, Harlak A, Yigit T, Balkan A, Balkan M, Cosar A (2008). Effect of intraoperative magnesium sulphate infusion on pain relief after laparoscopic cholecystectomy. Acta Anaesthesiol Scand..

[71] Louizos AA, Hadzilia SJ, Leandros E, Kouroukli IK, Georgiou LG, Bramis JP (2005). Postoperative pain relief after laparoscopic cholecystectomy: a placebo-controlled double-blind randomized trial of pre-incisional infiltration and intraperitoneal instillation of levobupivacaine 0.25%. Surg Endosc..

[72] Bisgaard T, Klarskov B, Kristiansen VB, Callesen T, Schulze S, Kehlet H (1999). Multi-regional local anaesthetic infiltration during laparoscopic cholecystectomy in patients receiving prophylactic multimodal analgesia: a randomized, double-blinded, placebo-controlled study. Anesth Analg..

[73] Pandey CK, Priye S, Ambesh SP, Singh S, Singh U, Singh PK (2006). Prophylactic gabapentin for prevention of postoperative nausea and vomiting in patients undergoing laparoscopic cholecystectomy: a randomized,double-blind, placebo-controlled study. J Postgrad Med..

[74] Tiippana E, Hamunen K, Kontinen V, Kalso E (2007). Do surgical patients benefit from perioperative gabapentin/pregabalin? A systematic review of efficacy and safety. Anesth Analg..

